# Contour Fillings

**Published:** 1869-06

**Authors:** C. R. Butler


### CONTOUR FILLINGS.

BY C. R. BUTLER, D. D. S., M. D.

Read before the Northern Ohio Dental Association.

*Mr. President and Gentlemen*:—At the last annual meeting of this association, it was your request that a paper be prepared and presented at this meeting on the question of contour fillings. We will first consider some of the elements and conditions that enter into the accomplishment of this object.

1st. The operator must be a man in the most complete sense, and he that has the greatest number of his physical and mental powers unimpaired, hereditarily or by accident, may, with favorable opportunities coupled with earnest toil, become proficient in any department of science and practice.

But just in degree as he lacks sphericity, there will be a want of contour and completeness in his work. Science as well as observation has taught us that the original form of the material world was a sphere. But as we look around and behold the terrible havoc and devastation, caused by volcanic inspiration and expiration, throwing the physical world into shapeless piles of rocks and mountains, interspersed with bottomless seas and seething whirlpools; can we wonder that man comes before us presenting so many deformities.

And, notwithstanding these seeming irreparable departures, there is an ever ceaseless struggle in the various departments of nature, to regain its pristine beauty, I trust you will pardon this seeming deviation for the purpose of illustration of the subject before us.

We as specialists are called upon to restore lost parts of the Dental organism, and we should ever be ambitious to use the noble faculties that the Great Father has bequeathed to us, not to be abused, but used to the utmost in the great work of restoring to health and beauty the organs we have to care for.

There is always a demand that keeps a little in advance of the supply, and it would not show health and vigor in any class of professional men, to be wanting in ability and disposition to meet promptly any legitimate demand made upon them. A few years ago the *demand* for contour operations was only occasional, and even then we were but partially able to meet the demand, for want of the proper knowledge and material.

But the progress that has been made in the past twelve years, shows that at least some have been equal to the demand.

We will briefly allude to some of the principal causes of the demand for contour fillings. The anatomical form and arrangement of the teeth for the performance of their functions in mastication and speech, is the strongest argument; and if any portion of the shape of the crown be destroyed, its usefulness and health is more or less impaired.

2d. The material that serves us best in the restoration of these lost parts is the adhesive gold foil, and the simple assertion of this fact, in any association would be apt to provoke discussion; and notwithstanding, I leave it before you on its merit, and class it as an all important element in contour fillings. Innumerable efforts have been made to substitute other materials, but with very limited success.

The preparation of a cavity for a restoration filling is a matter of some importance; in the first place, the extent of the margins should be brought to view, then they should be squared up smoothly as it were for the reception of a segment of the original crown; then form within the cavity as strong points for anchorage as possible, into which the gold must be securely packed, in order that the filling when completed, may stand the force of mastication without being displaced.

And in order that we may be able to make the most complete contour operations, we require improved forms of instruments, in order that the gold may be more thoroughly consolidated in its entire column or mass. But, some are ready to exclaim, you have not told us *how* you would go to work to make an operation in any individual case.

Supposable cases may be much easier cited than a real operation made, and yet the latter would teach far more. I will, however, give two or three cases in practice. A case of the approximal surfaces of the right inferior first and second molars, standing close together in the mouth of a girl, say twelve years old, timid and sensitive, and cavities small, shall we file. The mutilation of the teeth at such a point will not be seen every time the patient speaks or laughs, or shall we resort to the wedge; yes, even *here* it is far more preferable.

But can sufficient room be gained immediately, not easily; but if circumstances would permit, I would use a compressed hickory wedge, driving it between the teeth, not down upon the gum, and leave it a day; and then put in a piece of compressed pine, and keep it there a week or more, and the tenderness will nearly or quite subside.

On making the final operation, wedge them up firmly with orange wood by commencing to drive the wedge gently, until you have secured solidity; proceed to excavate and finish. A few days since I operated on such a case.

The next, a boy ten years old; approximal surfaces of the left superior second bicuspid and first molar. Secured space by immediate wedging; no break in the grinding surface from the side cavities. And the third, an adult, teeth firmly set, with approximal cavities in left inferior second bicuspid and first molar. Secured as much space as possible by wedging, then cut a groove from the grinding surface square down, filled and finished up in contour form. In all these cases there was quite a free flow of saliva.

Many cases of extensive restoration might be cited, and minutely described; but sufficient has been said to bring to your notice the all important advantage of contour operations in filling. The demand being upon us, and the ability within us, how can we be content to make imperfect fillings, or fail to see that we will ere long be laid aside as unworthy the great trust that has been committed to our keeping.

It is *impossible* to make *all* operations alike perfect. A man may be from various circumstances, such as fatigue, mental distraction, want of compatibility between patient and operator, or lack of time; a part or all of these causes may and will hinder the performance of a complete operation. Recognizing these modifying influences, we ought not to allow ourselves to be thus overborne.

The operator that persists in making operations day after day, and *uniformly* of a superior character, exhibits a higher degree of integrity than he that produces good fillings occasionally; or when all things are favorable, and the gold sticks every time just where he puts it.

I have known of cases where an extensive operation was demanded, and it required *days* for the operator to so order his forces, that the attempt would be a success instead of a failure.

Perhaps some are ready to say, operations that cost so much don't pay, and I am not willing to work unless it does. Well, the amputation of a broken leg, pays a finer fee for the time spent, than the tedious treatment of a fracture for the restoration of the disabled member. And yet, who is reckless enough to say, cut them off, because it is quicker done. I would not advocate a course of practice that would be at all likely to lead operators into difficulties of a pecuniary character.

The fact that some operators are better paid than others, is no argument why all should not be making earnest efforts to do equally well, fillings of ordinary size, which are in demand far in advance of any others.

There are some operators that are counted as good and reliable men, who either lack real skill or courage (perhaps both), to attempt operations of much magnitude with gold; and what is the effect? The tendency is for patients to think that Dr. A., is much more reasonable in his fees than Dr. B., who filled the same number of teeth for a person as did Dr. A., and charged twice or thrice as much. And who knows, but that Dr. A. received much the largest per cent. on services rendered.

Many will say, it takes too much time to make contour fillings, and I can't put in ten, twenty or more fillings in a day. Professional men generally, charge fees on the basis of time consumed, rather than quantity of work done. An artist that is ambitious to display his skill in the *number* of his pieces, rather than completeness of execution, rarely attains to a high degree of eminence in the artistic world.

If we are satisfied that contour fillings are better than the old styles of shapeless slabs and pegs, then why should we continue to reject them, any more than taking the old lumbering stage or canal route, instead of the beautiful modern steamer or Lightning railway train to New York.

I have said but little, detailing the manipulation in any class of fillings, for I have long since learned, to be understood or teach correctly, you must address the *eye* as well as ear.

In making any filling, especially contour, the case must be kept free from moisture; and to do this successfully with the least annoyance to patient, should be the determined effort of every operator. Some may ask, what class of fillings do you call *contour?* Approximal, buccal, label, lingual, or grinding surface. I answer, *all* fillings should be contour, gold packed into a cavity, and left either in excess or deficient; in one case it would well represent a scar or pockmark, and in the other an excrescence, rather than a contour operation.

